# Early Weaning Stress in Pigs Impairs Innate Mucosal Immune Responses to Enterotoxigenic *E. coli* Challenge and Exacerbates Intestinal Injury and Clinical Disease

**DOI:** 10.1371/journal.pone.0059838

**Published:** 2013-04-24

**Authors:** Brittney L. McLamb, Amelia J. Gibson, Elizabeth L. Overman, Chad Stahl, Adam J. Moeser

**Affiliations:** 1 Department of Population Health and Pathobiology, North Carolina State University College of Veterinary Medicine, Raleigh, North Carolina, United States of America; 2 Laboratory of Developmental Nutrition, Department of Animal Science, North Carolina State University, Raleigh, North Carolina, United States of America; 3 Center for Comparative Medicine and Translational Research (CCMTR), North Carolina State University, College of Veterinary Medicine, Raleigh, North Carolina, United States of America; Institut Jacques Monod - UMR 7592 CNRS - Université Paris Diderot, France

## Abstract

**Background and Aims:**

The clinical onset and severity of intestinal disorders in humans and animals can be profoundly impacted by early life stress. Here we investigated the impact of early weaning stress in pigs on intestinal physiology, clinical disease, and immune response to subsequent challenge with enterotoxigenic F18 *E. coli* (ETEC).

**Methodology:**

Pigs weaned from their dam at 16 d, 18 d, and 20 d of age were given a direct oral challenge of F18 ETEC at 26 d of age. Pigs were monitored from days 0 to 4 post-infection for clinical signs of disease. On Day 4 post-ETEC challenge, ileal barrier function, histopathologic and inflammatory cytokine analysis were performed on ileal mucosa.

**Results:**

Early weaned pigs (16 d and 18 d weaning age) exhibited a more rapid onset and severity of diarrhea and reductions in weight gain in response to ETEC challenge compared with late weaned pigs (20 d weaning age). ETEC challenge induced intestinal barrier injury in early weaned pigs, indicated by reductions in ileal transepithelial electrical resistance (TER) and elevated FD4 flux rates, in early weaned pig ileum but not in late weaned pigs. ETEC-induced marked elevations in IL-6 and IL-8, neutrophil recruitment, and mast cell activation in late-weaned pigs; these responses were attenuated in early weaned pigs. TNF levels elevated in ETEC challenged ileal mucosa from early weaned pigs but not in other weaning age groups.

**Conclusions:**

These data demonstrate the early weaning stress can profoundly alter subsequent immune and physiology responses and clinical outcomes to subsequent infectious pathogen challenge. Given the link between early life stress and gastrointestinal diseases of animals and humans, a more fundamental understanding of the mechanisms by which early life stress impacts subsequent pathophysiologic intestinal responses has implications for the prevention and management of important GI disorders in humans and animals.

## Introduction

Enteric infections resulting in diarrhea are a major cause of morbidity and mortality among both humans and agriculturally relevant animal populations worldwide. Enterotoxigenic *E. coli* (ETEC) are a major cause of these infections and are responsible for an estimated 300,000-5000 deaths in children under the age of 5[1]. These ETEC are also the leading agent associated with traveler’s diarrhea[2]and one of the leading causes of diarrhea in agricultural animals[3]. Knowledge regarding the molecular basis of intestinal infections has increased substantially over the past decade. It is known that ETEC colonizes the intestine and produces heat labile (LT), heat-stable (STa and STb), and enteroaggregative *E. coli* heat-stable enterotoxin-1 (EAST1) enterotoxins that trigger intracellular signaling pathways inducing massive fluid secretion that contribute to diarrhea. The LT toxin of ETEC binds to GM1(monosialotetrahexosylganglioside) ganglioside receptors on the enterocyte brush border leading to translocation, activation of adenylate cylase, and increases in cAMP-PKA pathways that induce phosphorylation-dependent increases in apical CFTR channel activity, resulting in Cl-, HC03- and fluid movement into the intestinal lumen[4], [5]. STa and EAST-1 enterotoxins induce fluid secretion via similar mechanisms although epithelial secretion pathways are mediated via cGMP [6].

It has become increasingly clear that environmental factors, including stress and diet, play a major role in gastrointestinal health and defense against important intestinal diseases in both humans and animals. Multiple lines of evidence derived from epidemiological and basic animal research studies show the important role of stress in the development and clinical onset of variety of debilitating gastrointestinal disorders including irritable bowel syndrome (IBS), the inflammatory bowel diseases (IBDs), and chronic intestinal infections[7], [8], [9], [10]. The mechanisms by which early life environmental stressors influence subsequent disease susceptibility and severity remain poorly understood. Our previous studies utilizing a porcine model of early weaning stress showed that early weaning (weaning <20 d of age) resulted in long-term deleterious changes in gastrointestinal barrier properties including impaired intestinal epithelial barrier function and increased inflammation [11]. To date, mechanistic studies in this porcine model and rodent models of early life stress have shown a central role for corticotropin releasing factor (CRF) receptor signaling pathways [11], [12], [13] and mast cell activation [14], [15] for immediate and long-term disturbances in intestinal epithelial barrier function. It is apparent that early life stress can have long-lasting effects on the immune system and intestinal barrier properties; however, the impact of early life stress on the intestinal response to subsequent infectious challenge has not been investigated previously. The objective of this study was to determine if early weaning stress in the pig alters subsequent intestinal injury and immune responses and clinical manifestations in response to an ETEC challenge.

## Materials and Methods

### Animals

All studies were approved by the North Carolina State University Institutional Animal Care and Use Committee. A total of 48 Large White × Landrace barrows, obtained from a commercial swine farm in North Carolina, were used in this study. Piglets selected for this study were genotyped at 1 week of age to confirm genetic susceptibility to F18 E coli based on the restriction fragment length polymorphism test described by Frydendahl et al.[16]Susceptible piglets were randomly assigned to 1 of 3 experimental groups based on the age at which they were weaned from the sow. The weaning age groups in this study included: 1) 16 d weaning age, 2) 18 d weaning age, and 3) 20 d weaning age. Each group contained n = 16 piglets. The weaning age groups were based on our previous studies demonstrated that early weaning (<20 days of age) resulting in long-lasting disturbances in intestinal barrier function, secretory activity, and immune cell activation compared with late weaned pigs (≥20 days of age) [11]. To reduce the potential confounding effects of parity and maternal immunity in this study, all piglets were obtained from the same farrowing group and piglets were selected such that sow parity was equally distributed among weaning age groups. All piglets were weaned from the sow, ear tagged, and transported to a North Carolina State University swine research facility for experimental studies. Weaned pigs were housed individually in climate controlled rooms. Pigs were offered *ad libitum* access to water and standard nursery diet that met or exceeded NRC recommendations (Swine NRC, 1998).

### Enterotoxigenic *E. coli* Challenge Experiments

Separate rooms were used to house control (non-infected) and infected pigs for the duration of the study. Pigs were challenged when they reached 26 days of age to ensure that all pigs were expressing F18 *E coli* receptor protein in the intestine[17]. Pigs received a direct oral challenge of 2×10∧9 CFU comprised of two strains of F18 ETEC as previously described [18].

Our rationale for studying F18 ETEC in this model is because of (1) our previous experience with this infection model[18] and (2) F18 ETEC induces comparable clinical disease, via common pathophysiologic mechanisms, in both animals and humans.

### Clinical Assessment

All pigs were weighed prior to *E. coli*. Challenge (Day 0) and at the end of the study (Day 4 post-challenge). Fecal scores were recorded daily throughout the experiment and were conducted by trained personnel that were blinded to experimental groups. Fecal scores were based on a standard scoring system (0, dry, hard, well-formed feces; 1, soft but formed feces; 2, pasty feces green or brown in color; 3, viscous feces in light color, episodic; 4, fluid feces in light color; 5, watery feces, continuous). Fecal scoring began on the day of arrival (Day -5 pre challenge) and continued for the duration of the study (Day 4). Body weights were recorded on Day 0 and Day 4 of the experiment to calculate the % body weight change over the 4 day experimental period.

### Necropsy and Gross Pathology

On Day 4 post infection (peak clinical disease), all pigs were euthanized by penetrating captive bolt, exsanguinated and a complete necropsy was performed to assess gross pathology including presence of fluid-filled intestines, edema, and hyperemia of abdominal organs.

### Ussing Chamber Studies

Distal small intestine (ileum) was harvested from each pig immediately after euthanasia and opened along the anti-mesenteric border. The intestinal mucosa was stripped from the seromuscular layer in oxygenated (95% O2- 5% CO2) Ringer solution (in mmol/l: 154 Na+, 6.3 K+, 137CL−, 0.3H2PO4, 1.2 Ca2+, 0.7 Mg2+, 24 HCO3−; pH 7.4) and mounted in 1.13 cm^2^aperture Ussing chambers (World Precision Instruments, Inc Sarasota, FL). Ileal mucosa was bathed on the serosal and mucosal sides with 10 ml Ringer solution. The serosal bathing solution contained 10 mM glucose, which was osmotically balanced on the mucosal side with 10 mMmannitol. Bathing solutions were oxygenated (95% O2- 5% CO2) and circulated in water-jacketed reservoirs maintained at 37°C. The spontaneous potential difference (PD) was measured using Ringer-agar bridges connected to calomel electrodes, and the PD was short-circuited through Ag-AgCl electrodes using a voltage clamp that corrected for fluid resistance. Tissues were maintained in the short-circuited state, except for brief intervals to record the open-circuit PD. Transepithelial electrical resistance (Ω⋅cm^2^) was calculated from the spontaneous PD and short-circuit current (*I*_sc_), as previously described[19]. After a 30-minute equilibration period on Ussing chambers, TER and *I*_sc_was recorded at 15-minute intervals over a 1-hour period and then averaged to derive the basal TER and *I*_sc_ values for a given animal.

### Paracellular Permeability to 4 kDaFITC Dextran (FD4)

After a 30-min equilibration period on Ussing chambers, FITC-albumin (Sigma, 100 mg/mL) was added to the mucosal bathing reservoir of Ussing chambers. After a 15 minute equilibration period, standards were taken from the serosal side of each chamberand a 60 minute flux period was established by taking 0.5 ml samples from the mucosal compartment. The quantity of FITC-albumin was established by measuring the fluorescence in mucosal reservoir fluid samples in a fluorescence plate reader at 540 nm. Data was presented as the rate of FD4 flux in mg FD4 flux.min.

### Histologic Analyses of Intestinal Tissues

Ileum was fixed in 10% neutral buffered formalin and processed for paraffin-embedding. Paraffin blocks were sectioned (5 µm thick) and stained with hematoxylin and eosin for histological analysis. Photomicrographs were acquired with 20×, 40× and 100× magnifications at a resolution using imaging software (Infinity Analyze Software (Lumenera, Ottawa, Ontario, Canada) running a high resolution digital camera (Lumenera, Ottawa, Ontario, Canada) equipped to a clinical light microscope (Meiji Microscope Solutions, Model OMFL400). Measurements for crypt depth and villous height were taken utilizing the calibrated measurement caliper option in the Villus measurements were taken from three well-oriented villi.Villi chosen for measurement were based on the criteria that 1) the entire crypt and villi were captured in cross section and 2) the central lacteal was present. Villi overlying gut associated lymphoid tissue was excluded from measurement.

The number of neutrophils was counted in histologic sections based on nuclear and cytoplasmic morphology. Neutrophil counts were performed at 40× magnification and counts were expressed as number of neutrophils/hpf.

### Mast Cell Counts and Activation

The number of mast cells and their activation status was performed as described previously [11] in porcine ileal tissues that were embedded and frozen in optimal cutting temperature medium (OCT, Tissue Tek). Frozen tissues were cut in 5 µm-thick sections and mounted on glass slides. Frozen sections of ileum (10 µm thick) were prepared and then fixed for 1 h in Carnoy’s fixative (60% ethanol-30% chloroform-10% glacial acetic acid). Sections were then stained for 45 min at room temperature with 0.5% toluidine blue in 0.5 N HCl in PBS. Sections were then viewed at ×20 objective, and mast cells were evaluated as either stableor degranulated. The critieria for stable mast cells included mast cells in which none to <10% of the mast cell granules were shown to be actively released around the cell whereas degranulated mast cells were assessed as mast cells which have released >50% of intracellular granules. Counts were conducted on five different fields per slide and 2 slides per treatment group. Data is presented as percentage of mast cells displaying each of the activity categories described above. All cell counts were performed a blinded reviewer who was blinded to experimental treatments.

### Ileal Cytokine Analysis

Ileal mucosa was homogenized in PBS containing protease inhibitors and the supernatant was collected and analyzed for protein content us a BCA assay[20]. Samples were then diluted 1;10 in PBS and assayed for TNFα, IL-8, and IL6 using a commercial porcine ELISA assays (R&D Systems, Minneapolis, MN). Concentrations of each cytokine were expressed on a per mg protein basis.

### Statistical Analyses

Data were reported as means±SE based on the experimental number (n). Time course data (fecal scores) were analyzed using a 2-way ANOVA on repeated measures with time and experimental group as the main factors. The remaining data were analyzed by using a standard one-way ANOVA (Sigmastat, Jandel Scientific, San Rafael, CA). A post hoc Tukey’s test was used to determine differences between treatments following ANOVA. Statistical significance was set at a level of p<0.05.

## Results

### Clinical Disease: Fecal Scores and Growth Rate

**Figure 1:**
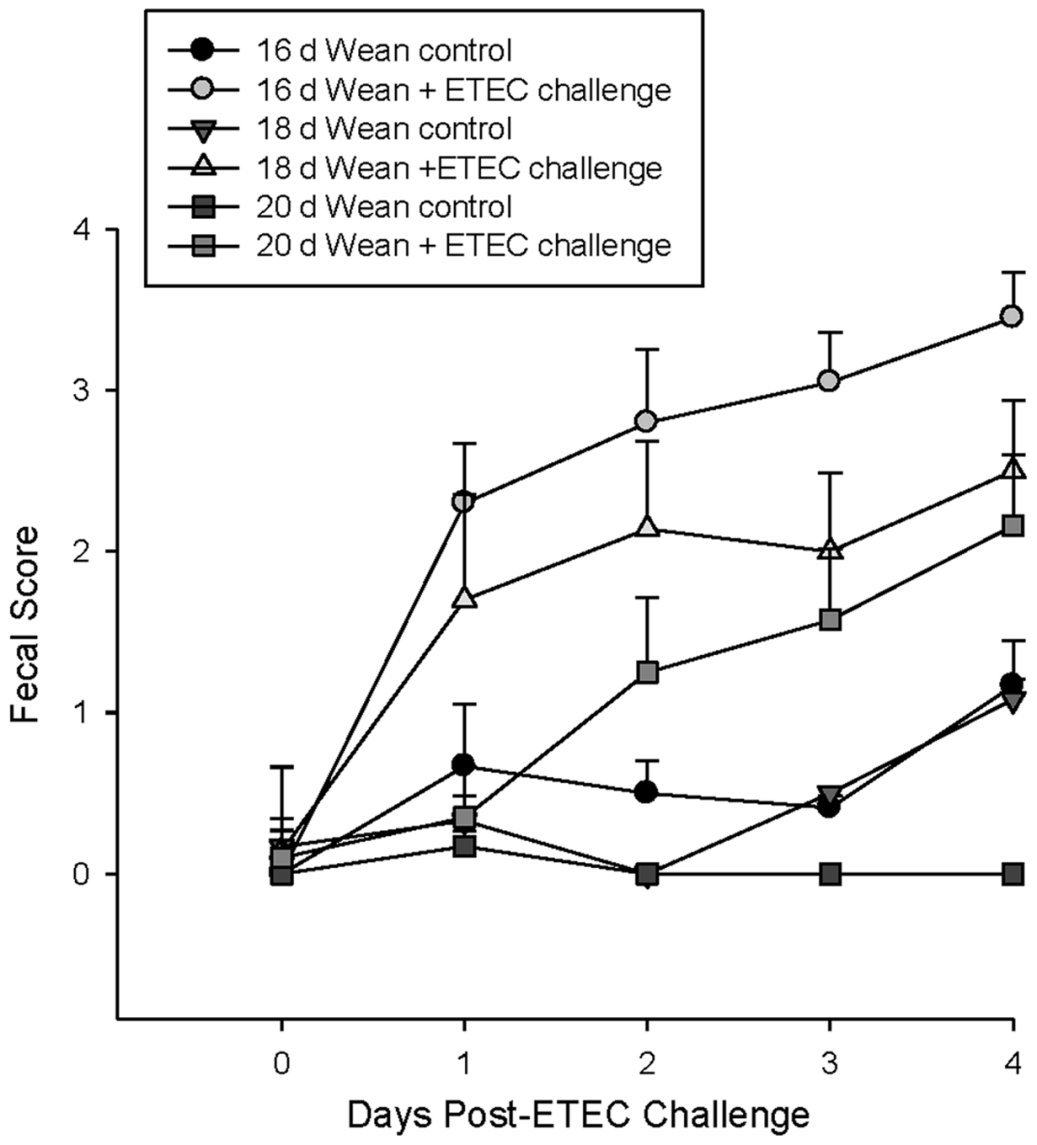
Effects of weaning age and ETEC challenge on fecal scores. Weaned pigs were challenged with ETEC (d 0) and fecal scores were recorded daily over a 4-d experimental period. Data represent means ±SE for n = 6−8 pigs/treatment group. Data were analyzed using a 2 way ANOVA on repeated measures with experimental group and time point post-challenge as the main variables. ETEC challenge increased fecal scores in all weaning age group. In ETEC challenged pigs, fecal scores were higher (P<0.05) in the 16 d weaning age group compared with 20 day weaning age group. Compared with controls, pigs in the 16 d and 18 d weaning age groups exhibited increased (P<0.001) fecal scores at earlier time points (on days 1 and 2 post-challenge) compared with 20 d weaning age group.

**Figure 2:**
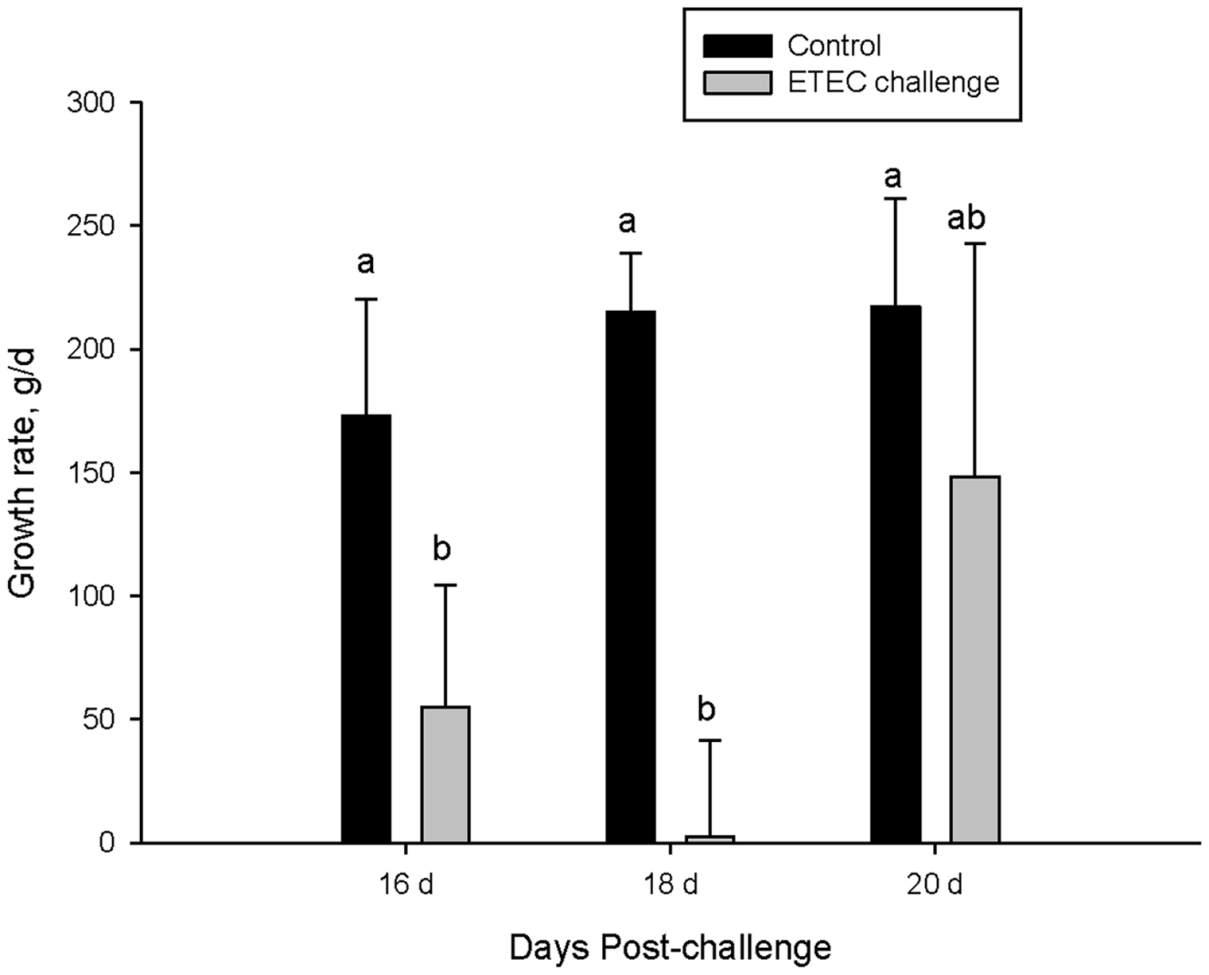
Influence of weaning age and ETEC challenge on growth rate in pigs. Data represent means ±SE for n = 6−8 pigs/treatment group. Data were analyzed using a T-test to compare differences between control and ETEC challenged pigs within earch weaning age group. Compared with controls, ETEC challenge reduced growth rate of pigs in the 16 d weaning age group (trend *P* = 0.06) and 18 d weaning age groups (P<0.05). ETEC challenge did not reduce growth rate in pigs weaned at 20 d of age.

Prior to ETEC challenge, there was no indication of diarrhea among any of the pigs in this study (Figure 1). Compared with controls, the ETEC challenge caused diarrhea (measured as increased fecal scores) in all weaning age groups (P<0.001); however, differences in the severity and time-of-onset were observed between weaning age groups. Diarrhea was first observed in the early weaning stress pig groups (16 d and 18 d weaning age groups) at 1-d post-ETEC challenge whereas pigs in the 20 d wean age group began to exhibit clinical diarrhea at 3 d post-challenge. Overall, diarrhea was more severe in pigs in the 16 d and 18 d weaning age groups indicated by higher (P<0.05) fecal scores compared with pigs in the 20 d weaning age group. ETEC challenge reduced growth rates in pigs weaned at 16 d (P = 0.06) and 18 d (P<0.05) over the 4-d challenge period; however, growth rate was not significantly affected in pigs weaned at 20 d of age (Figure 2).

### Ileal Barrier Function: Transpithelial Electrical Resistance (TER) and FD4 flux

**Figure 3:**
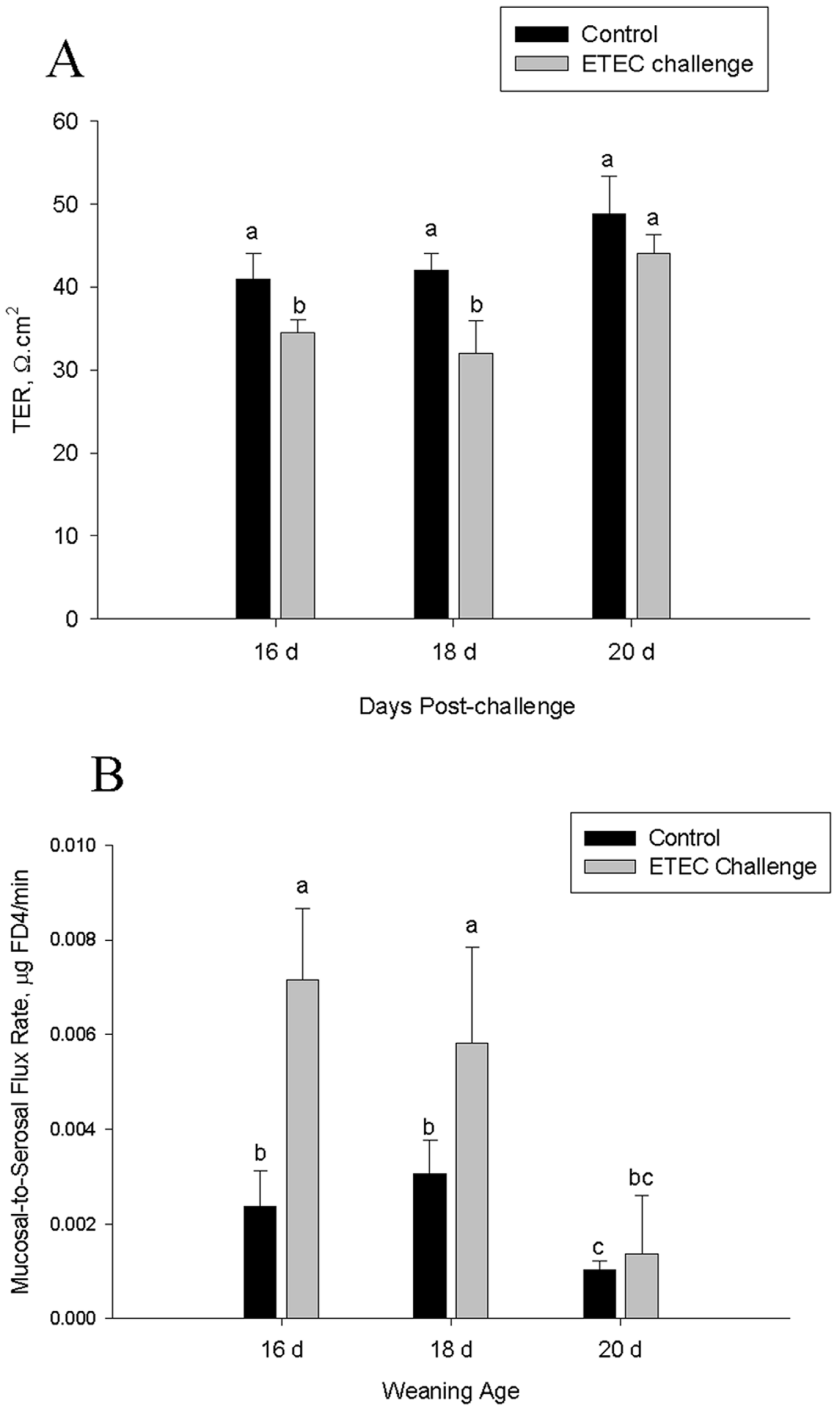
Influence of weaning age and ETEC challenge on ileal transepithelial electrical resistance (TER) and FITC dextran 4 kDa (FD4) flux. Data represent means ±SE for n = 8 pigs/treatment group. Data were analyzed using a 1-Way ANOVA, a Tukey’s test was performed to compare differences among treatment groups. Pigs weaned at 16d or 18 d of age and challenged with ETEC exhibited reduced Ileal TER (Panel A) and elevated FD4 flux (Panel B) compared with controls.

In control (non-challenged) pigs, ileal mucosal FD4 flux rates, an index of intestinal paracellular permeability, were elevated in the 16d and 18d weaning age pigs compared with pigs in the 20 d weaning age group(p<0.05; Figure 3B). ETEC challenge induced increases in intestinal permeability in early weaning stress pigs, indicated by reduced (P<0.05) TER (Figure 3A) and elevated (P<0.05) FD4 flux rates (Figure 3A). In contrast, there were no significant differences in ileal TER or FD4 flux rates between control and ETEC-challenged pigs weaned at 20 d of age.

### Ileal Short Circuit Current (*I_sc_*)

**Figure 4:**
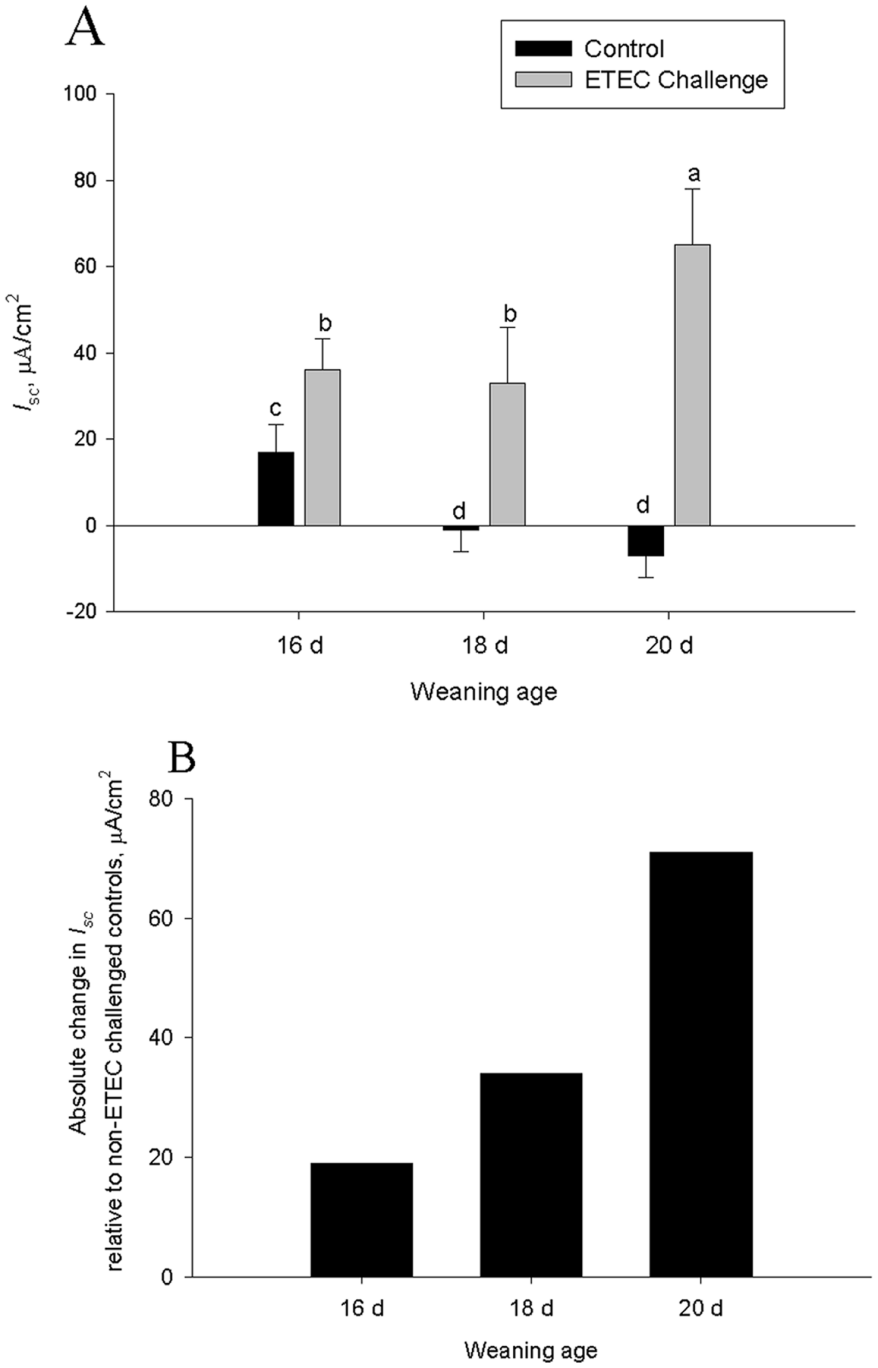
Influence of weaning age and ETEC challenge on ilealtransepithelial electrical resistance (TER) and FITC dextran 4 kDa (FD4) flux. Data represent means ±SE for n = 8 pigs/treatment group. Data were analyzed using a 1-Way ANOVA, a Tukey’s test was performed to compare differences among treatment groups. Ileum from all ETEC-challenged pigs exhibited greater *I*_sc_ compared with controls (Panel A). Ileum from pigs weaned at 20 d of age exhibited the greatest baseline *I*_sc_ values (Panel A) and absolute change in *I*_sc_ over controls (Panel B) compared with all other experimental groups.

Control (non-challenged) pigs weaned at 16 d of age exhibited greater baseline *I*_sc_, an index of electrogenic ion transport, compared with pigs weaned at 18 d or 20 d of age (Figure 4). In response to ETEC challenge, the greatest elevations in *I*_sc_ were observed in the ileum from pigs weaned at 20 d of age compared with ileum from other weaning age groups (Figure 4A). Given the different baseline *I*_sc_ values between weaning age groups, the magnitude of ETEC-induced *I*_sc_ was also expressed as an absolute change in *I*_sc_ (delta *I*_sc_) relative to group-matched, unchallenged controls (Figure 4B) and demonstrated an ETEC-induced increase in ileal *I*_sc_ by 19, 36, and 70 uA/cm^2^ in 16 d, 18 d, and 20 d weaning age groups, respectively.

### Ileal Morphological Analysis

**Figure 5:**
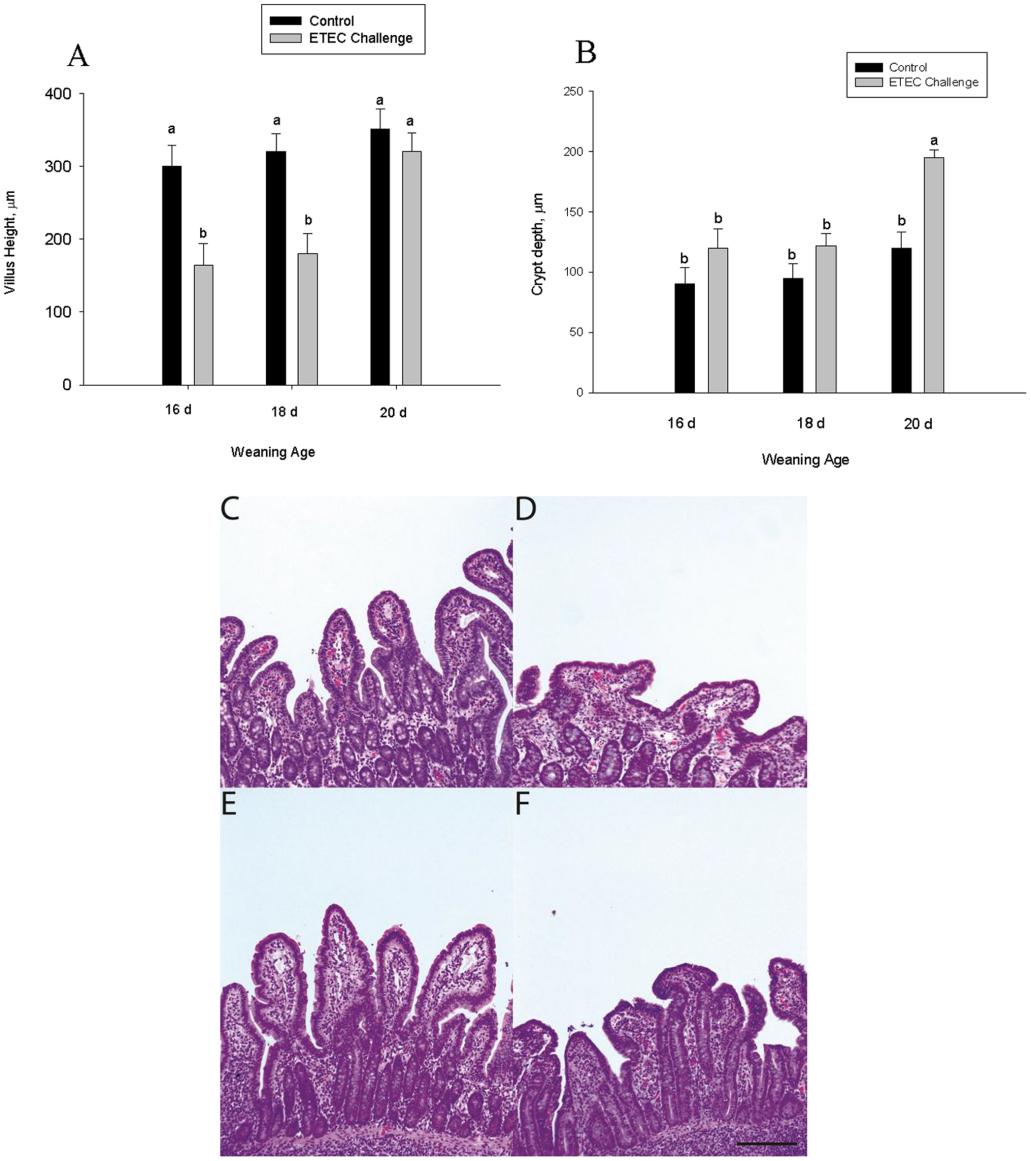
Influence of weaning age and ETEC challenge on villus height and crypt depth. Data represent means ±SE for n = 8 pigs/treatment group. Data were analyzed using a 1-Way ANOVA, a Tukey’s test was performed to compare differences among treatment groups.

In response to ETEC challenge, pigs in the 16 d and 18 d weaning age groups exhibited marked villus atrophy as indicated by reductions in villus height and morphological appearance of villi whereas no measureable changes in villus morphology were observed in ileum from challenged 20 d weaned pigs (Figure 5). There were no significant effects of ETEC challenge on crypt depth, an indirect marker of crypt cell proliferation, in pigs weaned at 16 or 18 d of age; however, a significant increase in crypt depth were observed in the 20 d weaning age group (Figure 5B).

### Mast Cell and Neutrophil Numbers in Ileal Mucosa

**Figure 6:**
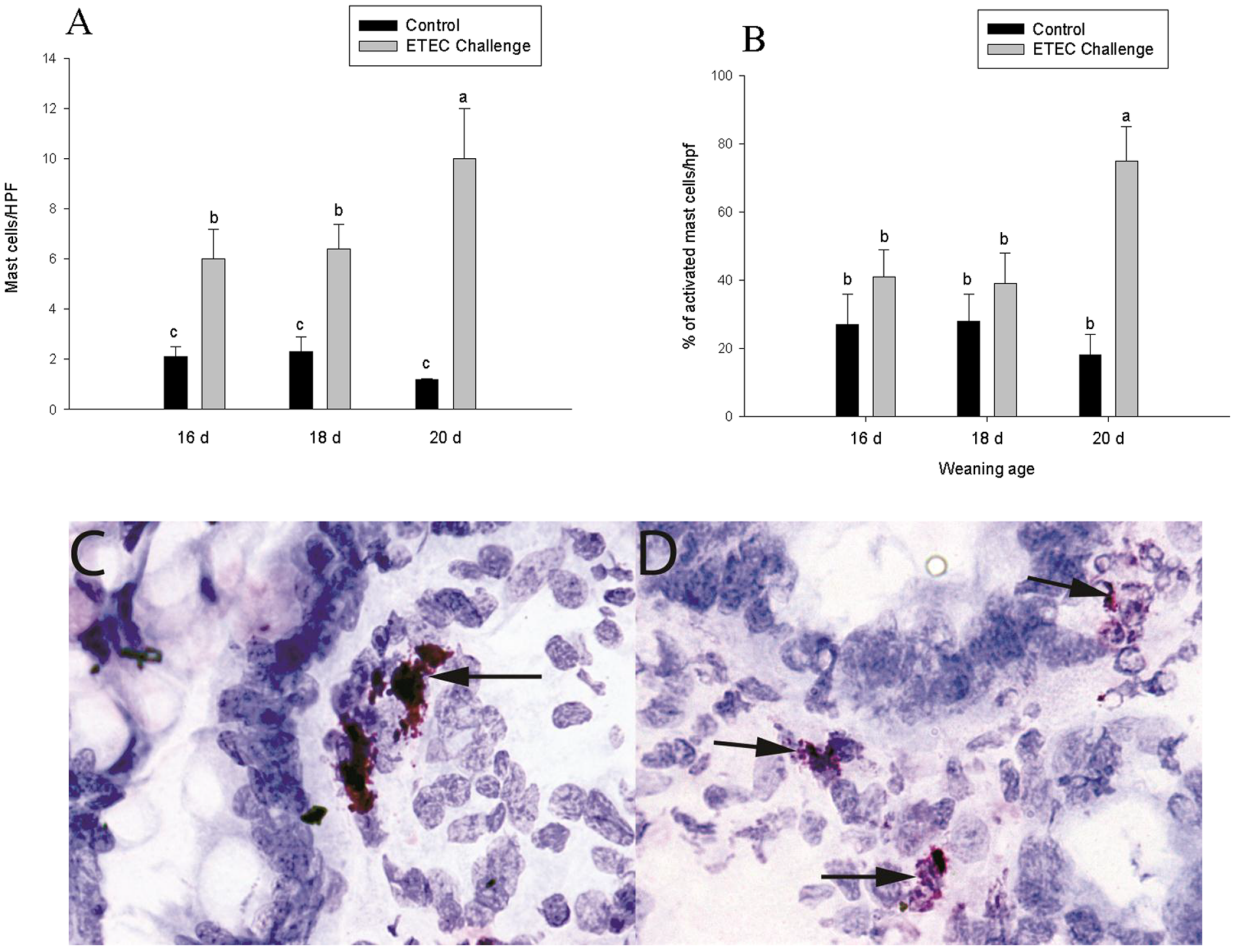
Influence of weaning age and ETEC challenge on mucosal mast cell numbers and activation. Values represent means ±SE for n = 8 ileal tissue sections/treatment group. Data were analyzed using a 1-Way ANOVA, a Tukey’s test was performed to compare differences among treatment groups. Increases in mucosal mast cell numbers (A) and activation (B) were observed in all weaning age groups; however pigs weaned at 20 d of age exhibited the greatest mast cell infiltration and activation compared with other weaning age groups. Images of ileal intestinal mucosa of ETEC-challenged early weaned pigs (16 d weaning age; Panel C) and late-weaned (20 d weaning age; Panel D). Mast cells in early weaned intestine are fully granulated and stain metachromatically (purple), whereas mast cells in late weaning intestine are degranulated indicated by loss of granularity and free purple granules in the surrounding tissue.

**Figure 7:**
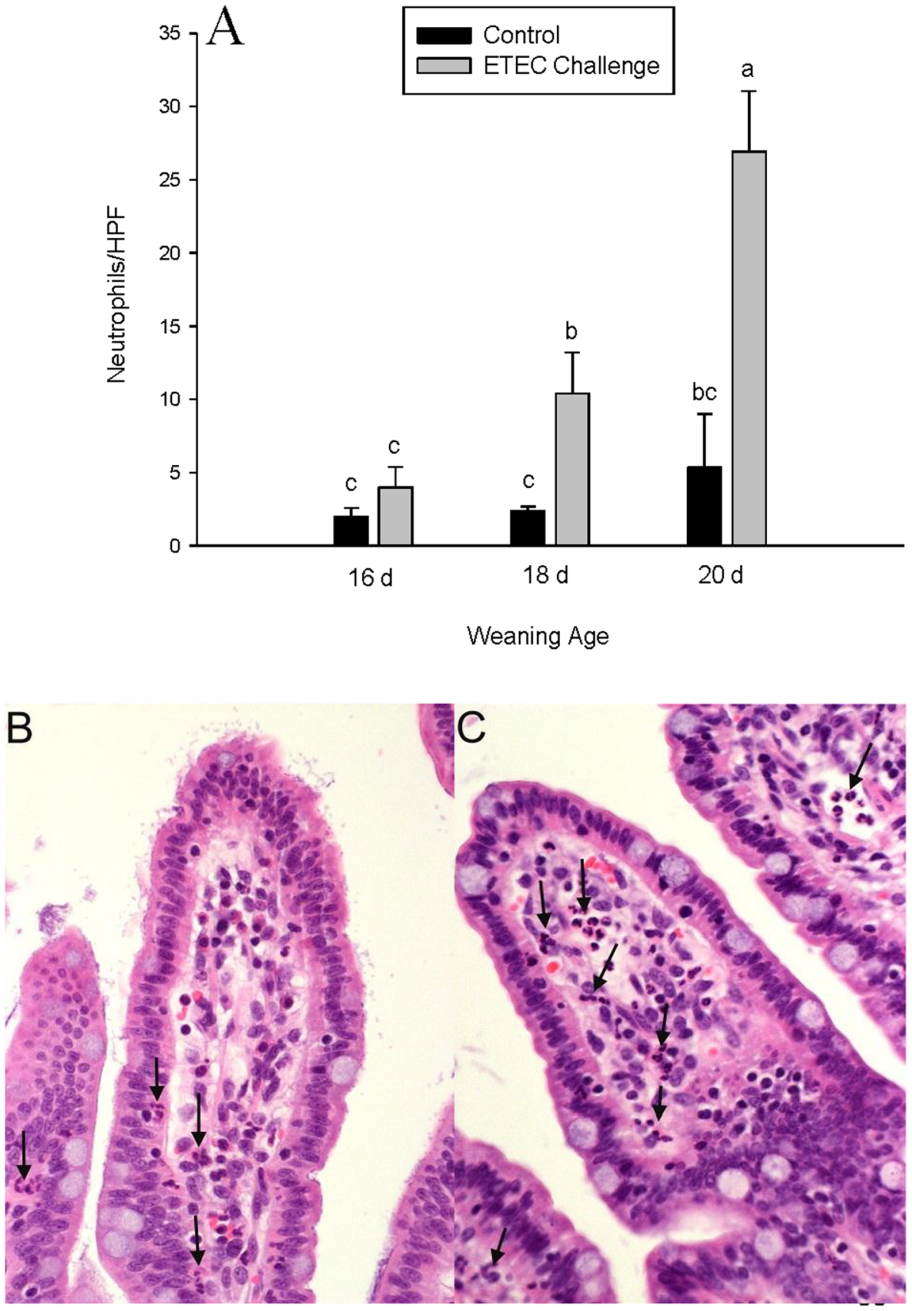
Effects of weaning age and ETEC challenge on neutrophil numbers in the porcine ileum. Values represent means ±SE for n = 8 ileal tissue sections/treatment group. Data were analyzed using a 1-Way ANOVA, a Tukey’s test was performed to compare differences among treatment groups.

Increased numbers of mast cells were observed in the lamina propria of pigs challenged with ETEC however; this response was significant greater in pigs weaned at 20 d of age (Figure 6A). Histological analysis of mast cells also revealed a marked increase in the percentage of degranulated mast cells in ETEC-challenged pigs weaned at 20 d of age compared with other weaning age groups (Figure 6B and C). Ileal mucosal neutrophil numbers were similar between control and ETEC challenged pigs weaned at 16 d of age and were slightly elevated (p<0.05)in pigs 18 d of age. In contrast, ileum from pigs weaned at 20 d of age exhibited an increase in neutrophil numbers in ETEC challenged (6.7-fold increase). (Figure 7).

### Ileal Cytokine Levels

**Figure 8:**
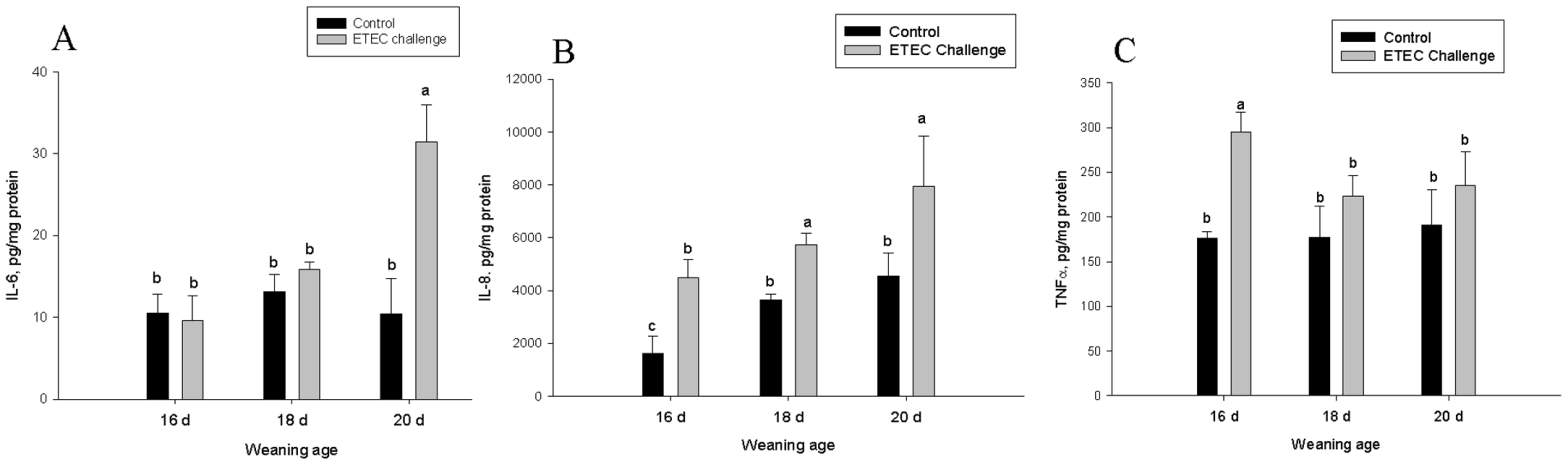
Influence of weaning age and ETEC challenge on ileal mucosal TNFα, IL-6, and IL-8. Values represent means ± SE, n = 8.Means without a common letter differ, *P*<0.05. TNFα levels in ileal mucosa were increased by ETEC challenge in pigs weaned at 16 d of age age but not 18 d or 20 d of age. IL-6 levels were elevated by ETEC challenge only in pigs weaned at 18 d of age. IL-8 levels were elevated by ETEC challenge in all weaning age group; however, the highest IL-8 levels were observed in ileum from pigs weaned at 20 d of age.

The concentration of inflammatory cytokines IL-6, IL-8, and TNF-α were determined in ileal mucosal samples (Figure 8). Ileal TNF-α levels were elevated (by 1.8-fold) in ETEC-challenged pigs that were weaned at 16 d of age, but no increases in TNF-α were observed in pigs weaned at 18 and 20d of age. A 3-fold increase in IL-6 was observed in ETEC-challenged pigs in the 20 d weaning age group; however, no increases were observed in other challenged weaning age groups. IL-8 was elevated in all weaning age groups challenged with ETEC; however, the greatest IL-8 concentrations were observed in pigs weaned at 20 d of age.

## Discussion

Emerging evidence indicates that the the clinical onset and severity of a number of acute and chronic intestinal diseases, is profoundly and negatively influenced by early life stress [21], [22], [23]. Our previous studies have demonstrated that early weaning stress in the pig induces immediate and long-term deleterious effects on intestinal defense mechanisms including lasting disturbances in intestinal barrier function (increased intestinal permeability), increased electrogenic ion transport, and dysregulated intestinal immune activation.[11], [14] The mechanisms by which early life stress induces long-lasting disturbances in gut function are not fully understood; however, it has been demonstrated in rodent and porcine models that key signaling pathways by which early life stress alters intestinal function involve interplay between corticotropin releasing factor (CRF) system, mast cells, and enteric nerves.[11], [12], [15], [24]. While it is evident that early weaning and/or early life stress has significant and long-lasting impacts on intestinal function, its impact on the pathophysiologic response to subsequent intestinal infections have not been studied. The objective of the present study was to determine the impact of early weaning stress in pigs on the pathophysiologic and clinical responses to later intestinal infectious challenges. Results from this study demonstrated that pigs subjected to early weaning stress exhibited a more rapid onset and severe diarrhea and more rpfound reductions in growth rate, compared with late-weaned pigs. Furthermore, exacerbated ETEC-mediated clinical disease in early weaning stress pigs coincided with more pronounced histopathological intestinal injury and disturbances in intestinal barrier function (increased permeability) and electrogenic ion transport.

In response to an infectious challenge, the primary goal of the innate immune response is to rapidly clear or contain offending pathogens to prevent prolonged inflammation and sepsis [25]. The innate immune response is initiated by the recognition of bacterial ligands and activation of epithelial and resident sub-epithelial immune cells, such as mast cells[26], [27], macrophages[28] and dendritic cells[29], resulting in a rapid burst of pro-inflammatory cytokines (e.g. IL-6, IL-8, and TNF-α) and lipid-derived mediators (e.g. prostaglandins, leukotrienes) into the surrounding tissue and circulation. Released pro-inflammatory mediators recruit effector cells such as neutrophils to the site of infection where, via multiple mechanisms, they aid in containing and eventually clearing the pathogen. The critical importance of this response has been demonstrated in infection and sepsis models in which animals lacking key innate immune functions exhibit decreased bacterial clearance and increased clinical disease and mortality [30], [31], [32]. The importance of innate immune responses in the pig to ETEC infection has been demonstrated by Loos et al (2012) who, utilizing a small intestinal perfusion technique, showed that several innate immune genes were up-regulated by ETEC infection including IL1B IL17A,MMP3, PAP, IL8, and MMP1 [33]. In the present study, we hypothesized that an impaired innate immune response in early weaning stress pigs contributed to the exacerbated disease in response to ETEC challenge. In line with this hypothesis, our data demonstrate that ETEC challenge induced a robust innate immune response in late weaned pigs indicated by marked elevations in IL-6 and IL-8 and recruitment of neutrophils to the intestine; however, this response was markedly attenuated in early weaning stress pigs. In contrast, at 4 d post ETEC challenge, elevated TNF level were only detected in ETEC-challenged, early weaning stress pigs. The precise functional relationship between the differential cytokine responses in early weaned pigs and exacerbated clinical disease in this study is presently unclear; however, the role of these cytokines in bacterial defense and clearance and host survival has been investigated previously. It is known that IL-6, is a pleiotropic cytokine that is produced by a number of cell types including macrophages, endothelial cells, B cells and mast cells, plays a crtical role in the host response to infection and inflammation[34], [35]. The protective role of IL-6 has been demonstrated in IL6−/− mice which exhibit higher mortality when infected with various pathogens including *E. coli* [36], *Klebsiella pneumonia* [37], or *Streptococcus pneumonia* [38]. In contrast, blockade of IL-6 activity with IL-6 antibodies improves survival in a polymicrobial peritoneal sepsis model [39]. IL-6 was shown to be protective by enhancing neutrophil chemotaxis and killing. IL-8, another crtical mediator in the innate immune response to infection, is produced by several cell typescells and has been shown to be largely responsible for neutrophil recruitment to infected sites [40], [41]. Given the protective role of IL-6 and IL-8, results from the present study suggest that the suppressed IL-6, IL8, and cellular inflammatory response observed in ETEC-challenged early weaning stress pigs, may have contributed to increased intestinal injury and clinical disease. As mentioned above, TNF levels were elevated in ETEC infected early weaned pigs compared with late-weaned pigs. In support of these findings, O’Mahony et al (2009) demonstrated that *in vitro* stimulation of whole blood with LPS from rats subjected to maternal separation stress induced an increase in TNF release compared with control (normal reared animals)[8]; however, in contrast to the present study, the authors did not observe differences in LPS-induced IL-6;,this could be due to species differences or differences between systemic and local immune responses.

Intestinal mast cells play a central role in innate immune response to bacterial, parasitic, and viral infections by releasing pro-inflammatory cytokines (TNF, IL-6, LTB4) that mediate neutrophil recruitment into infected tissues and bacterial clearance [42], [43]. Specifically, it has been shown that mast cell-derived IL-6 is a major mediator of survival from severe infections by enhancing intracellular killing of bacteria by neutrophils [44]. There is a paucity of data regarding influence of weaning age or early life stress on innate mast cell responses to subsequent infections. Given that (1) mast cells play a central role in the innate immune response to enteric infections by releasing mediators that recruit neutrophils and (2) early weaned pigs exhibited suppressed cytokine production and neutrophil recruitment in the present study, we investigated whether early weaning stress impacted intestinal mast cell activation in ETEC-challenged pigs. Our studies revealed that while ETEC-induced increases in mast cell numbers and marked degranulation in late-weaned control pigs, mast cell degranulation was profoundly attenuated in early weaned pigs. The precise role of suppressed mast cell activation in disease exacerbation in ETEC-challenged, early weaned pigs in this study is not known; however, it is plausible that this could represent an important mechanism for diminished cytokine responses and neutrophil infiltration and exacerbated clinical disease observed in early weaned pigs. With regards to elevated TNF levels and impaired mast cell degranulation observed in early weaning stressed pigs, Piliponsky et al., (2012) demonstrated in a cecal ligation and puncture model of sepsis that TNF levels could be negatively regulated via degradation by mast cell chymase [45]. Therefore, impaired mast cell degranulation in ETEC-challenged early weaned pigs could have contributed to elevated TNF as a result of reduced degradation by mast cell proteases; however, this will need further investigations to confirm this relationship. Overall, the present study and our previous studies demonstrate that mast cells play a key role in stress-induced intestinal disturbances; however, the mechanisms by which early life stress impacts mast cell function remains poorly understood. Our previous investigations demonstrated that early weaning stress in the pig induces a chronic, low grade mast cell degranulation that is responsible for persistent epithelial barrier disturbances [11]. In the present study, we confirmed the increased baseline mast cell degranulation in early weaned pigs; however, when early weaned pigs were challenged with ETEC, the mast cell degranulation response was significantly impaired. These apparent divergent findings with regards to mast cell activation may suggest that while early weaning stress leads to chronic low-grade degranulation and intestinal barrier injury, innate mast cell responses to acute host infections may be significantly compromised and contribute to susceptibility and enhanced severity of infectious diarrheal disease., overall suggesting that specific mast cell signaling pathways (neuroendocrine stress-induced degranulation vs. pathogen-mediated immune functions) are differentially impacted by early life stress.

In the present study, early weaning led to marked differences in the intestinal pathophysiologic response to ETEC challenge. While late weaned pigs exhibited no measurable intestinal barrier injury in response to ETEC challenge (based on TER and FD4 flux measurements), intestinal barrier function was in challenged, early weaned pigs was significant. The differential responses to ETEC challenge were independent of age and feed intake as all piglets were challenged at 26 d of age and feed intake was similar between weaning age group during the experiment. The diminished ETEC-mediated inflammatory response observed in early weaned pigs does not support a major role for inflammatory signaling pathways in mediating intestinal barrier injury in this study. It is well-established that ETEC mediates its pathogenesis via the binding to receptors expressed on the intestinal epithelium. Once bound, ETEC elaborates heat stable (STa and STb) and heat labile enterotoxins (LT) that stimulate epithelial secretory signaling pathways resulting in massive Cl^-^, HCO_3_- secretion and fluid loss that is responsible for the clinical signs of diarrhea [4], [5], [46]. Although heat stable and heat labile toxins of ETEC are not know to induce direct epithelial damage, excessive fluid loss can result in hypovolemia contributing to ischemic conditions in the intestine that initiate profound defects in barrier function and villus structure which could contribute significantly to barrier injury and villus atrophy observed in this study. Furthermore, in severe ETEC infections, especially in neonates, intestinal barrier and morphological injury can be compounded by subsequent septicemia and multiple organ dysfunction [46].

Compared with late-weaned pigs, early weaned pigs exhibited diminished *I*_sc_ responses to ETEC challenge (by 3.7 and 2.1-fold compared with late-weaned pigs), yet exhibited increased diarrheal scores. This finding was surprising given that *I*_sc_ is reflective of net electrogenic ion transport, associated with Cl^-^ and HCO_3_^-^ secretion, which drives fluid movement into the intestinal lumen resulting in diarrhea. The enhanced secretory response to ETEC challenge, along with the robust innate immune response observed in the late-weaned pig ileum, could represent an enhanced capacity of the late-weaned pig to rapidly “flush-out’ and clear the offending pathogen and thus reduce clinical disease. However, it remains unclear why enhanced electrogenic *I*_sc_ responses in the late weaned ileum, was associated with less severe clinical diarrhea. One potential explanation could be due to enhanced colonic reabsorption of ETEC-stimulated ileal fluid in the late-weaned pig. In previous studies in pigs infected with TGE, it was shown that survival and reduced clinical diarrhea in older pigs (compared with neonates) was due to the more developed colonic microbiota and short chain fatty acid production that was responsible for compensatory fluid reabsorption in the colon [47]. In the present study, pigs were challenged with ETEC at the same age therefore, age related effects did not appear to be relevant in this study. The influence of early weaning stress on long-term colonic physiology and microbiota has not been studied in detail; however, evidence from rodent[8] and primate studies[48] indicate early life stress can impact development of the microbiota and potentially influence fluid absorptive capacity in the colon.

Overall these data demonstrate that early weaning stress in pigs has a profound impact on subsequent clinical severity and intestinal injury in response to ETEC challenge. The exacerbated clinical and pathophysiological consequences of early weaning stress in pigs coincided with defects in intestinal epithelial barrier function and suppressed mucosal innate innate immune responses that were possibly mediated via alterations in mast cell function. Given the emerging relationship between early life stress and subsequent gastrointestinal disease susceptibility in humans, and the central role of impaired intestinal epithelial and innate immune defense barriers in the onset of such diseases, a more fundamental understanding of the precise mechanisms by which early life stress compromises long-term intestinal defense mechanisms could lead to innovative management and therapeutic strategies for a number of important GI diseases of humans and animals.

## References

[1] WHO (2006) Future directions for research on enterotoxigenic Escherichia coli vaccines for developing countries. Wkly Epidemiol Rec 81: 97–104. 16671213

[2] Hill DR, Beeching NJ (2010) Travelers’ diarrhea. Current Opinion in Infectious Diseases 23: 481–487. 20683261 10.1097/QCO.0b013e32833dfca5

[3] Nagy B, Fekete PZ (2005) Enterotoxigenic Escherichia coli in veterinary medicine. Int J Med Microbiol 295: 443–454. 16238018 10.1016/j.ijmm.2005.07.003

[4] Field M (2003) Intestinal ion transport and the pathophysiology of diarrhea. J Clin Invest 111: 931–943. 12671039 10.1172/JCI18326 PMC152597

[5] Sack RB, Gorbach SL, Banwell JG, Jacobs B, Chatterjee BD, et al (1971) Enterotoxigenic Escherichia coli isolated from patients with severe cholera-like disease. J Infect Dis 123: 378–385. 4938945 10.1093/infdis/123.4.378

[6] Moeser AJ, Blikslager AT (2007) Mechanisms of porcine diarrheal disease. J Am Vet Med Assoc 231: 56–67. 17605665 10.2460/javma.231.1.56

[7] Dapoigny M (2009) Irritable bowel syndrome: epidemiology/economic burden. Gastroenterol Clin Biol 33 Suppl 1S3–8. 19303536 10.1016/S0399-8320(09)71519-2

[8] O’Mahony SM, Marchesi JR, Scully P, Codling C, Ceolho AM, et al.. (2008) Early Life Stress Alters Behavior, Immunity, and Microbiota in Rats: Implications for Irritable Bowel Syndrome and Psychiatric Illnesses. Biol Psychiatry. 10.1016/j.biopsych.2008.06.026 18723164

[9] Vaiserman A (2011) Early-life origin of adult disease: evidence from natural experiments. Exp Gerontol 46: 189–192. 20833239 10.1016/j.exger.2010.08.031

[10] Agostini A, Rizzello F, Ravegnani G, Gionchetti P, Tambasco R, et al (2010) Adult attachment and early parental experiences in patients with Crohn’s disease. Psychosomatics 51: 208–215. 20484718 10.1176/appi.psy.51.3.208

[11] Smith F, Clark JE, Overman BL, Tozel CC, Huang JH, et al (2010) Early weaning stress impairs development of mucosal barrier function in the porcine intestine. Am J Physiol Gastrointest Liver Physiol 298: G352–363. 19926814 10.1152/ajpgi.00081.2009 PMC2838512

[12] Moeser AJ, Klok CV, Ryan KA, Wooten JG, Little D, et al (2007) Stress signaling pathways activated by weaning mediate intestinal dysfunction in the pig. Am J Physiol Gastrointest Liver Physiol 292: G173–181. 16901995 10.1152/ajpgi.00197.2006

[13] Gareau MG, Jury J, Perdue MH (2007) Neonatal maternal separation of rat pups results in abnormal cholinergic regulation of epithelial permeability. Am J Physiol Gastrointest Liver Physiol 293: G198–203. 17510196 10.1152/ajpgi.00392.2006

[14] Moeser AJ, Ryan KA, Nighot PK, Blikslager AT (2007) Gastrointestinal dysfunction induced by early weaning is attenuated by delayed weaning and mast cell blockade in pigs. Am J Physiol Gastrointest Liver Physiol 293: G413–421. 17525151 10.1152/ajpgi.00304.2006

[15] Barreau F, Salvador-Cartier C, Houdeau E, Bueno L, Fioramonti J (2008) Long-term alterations of colonic nerve-mast cell interactions induced by neonatal maternal deprivation in rats. Gut 57: 582–590. 18194988 10.1136/gut.2007.126680

[16] Frydendahl K, Kare Jensen T, Strodl Andersen J, Fredholm M, Evans G (2003) Association between the porcine Escherichia coli F18 receptor genotype and phenotype and susceptibility to colonisation and postweaning diarrhoea caused by E. coli O138:F18. Vet Microbiol 93: 39–51. 12591205 10.1016/s0378-1135(02)00348-6

[17] Coddens A, Verdonck F, Tiels P, Rasschaert K, Goddeeris BM, et al (2007) The age-dependent expression of the F18+ E. coli receptor on porcine gut epithelial cells is positively correlated with the presence of histo-blood group antigens. Vet Microbiol 122: 332–341. 17353102 10.1016/j.vetmic.2007.02.007

[18] Cutler SA, Lonergan SM, Cornick N, Johnson AK, Stahl CH (2007) Dietary inclusion of colicin e1 is effective in preventing postweaning diarrhea caused by F18-positive Escherichia coli in pigs. Antimicrob Agents Chemother 51: 3830–3835. 17724148 10.1128/AAC.00360-07 PMC2151407

[19] Argenzio RA, Liacos JA (1990) Endogenous prostanoids control ion transport across neonatal porcine ileum in vitro. AmJVetRes 51: 747. 2337271

[20] Peace RM, Campbell J, Polo J, Crenshaw J, Russell L, et al (2011) Spray-Dried Porcine Plasma Influences Intestinal Barrier Function, Inflammation, and Diarrhea in Weaned Pigs. Journal of Nutrition 141: 1312–1317. 21613450 10.3945/jn.110.136796

[21] Agostini A, Rizzello F, Ravegnani G, Gionchetti P, Tambasco R, et al (2010) Parental bonding and inflammatory bowel disease. Psychosomatics 51: 14–21. 20118436 10.1176/appi.psy.51.1.14

[22] Lerebours E, Gower-Rousseau C, Merle V, Brazier F, Debeugny S, et al (2007) Stressful life events as a risk factor for inflammatory bowel disease onset: A population-based case-control study. Am J Gastroenterol 102: 122–131. 17100973 10.1111/j.1572-0241.2006.00931.x

[23] Naliboff BD, Kim SE, Bolus R, Bernstein CN, Mayer EA, et al.. (2011) Gastrointestinal and Psychological Mediators of Health-Related Quality of Life in IBS and IBD: A Structural Equation Modeling Analysis. Am J Gastroenterol. 10.1038/ajg.2011.377 PMC3855477 22085819

[24] Santos J, Saunders PR, Hanssen NP, Yang PC, Yates D, et al (1999) Corticotropin-releasing hormone mimics stress-induced colonic epithelial pathophysiology in the rat. Am J Physiol 277: G391–399. 10444454 10.1152/ajpgi.1999.277.2.G391

[25] Medzhitov R, Janeway CA Jr (1997) Innate immunity: impact on the adaptive immune response. Curr Opin Immunol 9: 4–9. 9039775 10.1016/s0952-7915(97)80152-5

[26] McLachlan JB, Hart JP, Pizzo SV, Shelburne CP, Staats HF, et al (2003) Mast cell-derived tumor necrosis factor induces hypertrophy of draining lymph nodes during infection. Nature immunology 4: 1199–1205. 14595438 10.1038/ni1005

[27] Malaviya R, Ross E, Jakschik BA, Abraham SN (1994) Mast cell degranulation induced by type 1 fimbriated Escherichia coli in mice. The Journal of clinical investigation 93: 1645–1653. 7512987 10.1172/JCI117146 PMC294203

[28] van der Windt GJ, van ’t Veer C, Florquin S, van der Poll T (2010) CD44 deficiency is associated with enhanced Escherichia coli-induced proinflammatory cytokine and chemokine release by peritoneal macrophages. Infection and Immunity 78: 115–124. 19901064 10.1128/IAI.00949-09 PMC2798194

[29] Qian C, Cao X (2012) Naturally occurring CD1c+ human regulatory dendritic cells: immunoregulators that are expanded in response to E. coli infection. European journal of immunology 42: 1388–1392. 22678895 10.1002/eji.201242632

[30] Malaviya R, Ikeda T, Ross E, Abraham SN (1996) Mast cell modulation of neutrophil influx and bacterial clearance at sites of infection through TNF-alpha. Nature 381: 77–80. 8609993 10.1038/381077a0

[31] Sutherland RE, Olsen JS, McKinstry A, Villalta SA, Wolters PJ (2008) Mast cell IL-6 improves survival from Klebsiella pneumonia and sepsis by enhancing neutrophil killing. J Immunol 181: 5598–5605. 18832718 10.4049/jimmunol.181.8.5598 PMC2610024

[32] Belaaouaj A, McCarthy R, Baumann M, Gao Z, Ley TJ, et al (1998) Mice lacking neutrophil elastase reveal impaired host defense against gram negative bacterial sepsis. Nat Med 4: 615–618. 9585238 10.1038/nm0598-615

[33] Loos M, Geens M, Schauvliege S, Gasthuys F, van der Meulen J, et al (2012) Role of heat-stable enterotoxins in the induction of early immune responses in piglets after infection with enterotoxigenic Escherichia coli. Plos One 7: e41041. 22815904 10.1371/journal.pone.0041041 PMC3398878

[34] Akira S, Taga T, Kishimoto T (1993) IL-6 in biology and medicine. Adv Immunol 54: 1. 8379461 10.1016/s0065-2776(08)60532-5

[35] Naka T, Narazaki M, Hirata M, Matsumoto T, Minamoto S, et al (1997) Structure and function of a new STAT-induced STAT inhibitor. Nature 387: 924–929. 9202127 10.1038/43219

[36] Dalrymple SA, Slattery R, Aud DM, Krishna M, Lucian LA, et al (1996) Interleukin-6 is required for a protective immune response to systemic Escherichia coli infection. Infection and Immunity 64: 3231–3235. 8757858 10.1128/iai.64.8.3231-3235.1996 PMC174212

[37] van Enckevort FHJ, Sweep CGJ, Span PN, Netea MG, Hermus A, et al (2001) Reduced adrenal response and increased mortality after systemic Klebsiella pneumoniae infection in interleukin-6-deficient mice. European Cytokine Network 12: 581–586. 11781184

[38] vanderPoll T, Keogh CV, Guirao X, Buurman WA, Kopf M, et al (1997) Interleukin-6 gene-deficient mice show impaired defense against pneumococcal pneumonia. Journal of Infectious Diseases 176: 439–444. 9237710 10.1086/514062

[39] Riedemann NC, Guo RF, Ward PA (2003) Novel strategies for the treatment of sepsis. Nature Medicine 9: 517–524. 10.1038/nm0503-517 12724763

[40] Agace WW, Patarroyo M, Svensson M, Carlemalm E, Svanborg C (1995) Escherichia-coli induces transuroepithelial neutrophil migration by an intercellular-adhesion molecule-1-dependent mechanisms. Infection and Immunity 63: 4054–4062. 7558319 10.1128/iai.63.10.4054-4062.1995 PMC173570

[41] Ko YC, Mukaida N, Ishiyama S, Tokue A, Kawai T, et al (1993) Elevated interleukin-8 levels in the uring of patients with urinary-tract infections. Infection and Immunity 61: 1307–1314. 8454332 10.1128/iai.61.4.1307-1314.1993 PMC281363

[42] Abraham SN, St John AL (2010) Mast cell-orchestrated immunity to pathogens. Nat Rev Immunol 10: 440–452. 20498670 10.1038/nri2782 PMC4469150

[43] Bischoff SC (2009) Physiological and pathophysiological functions of intestinal mast cells. Semin Immunopathol 31: 185–205. 19533134 10.1007/s00281-009-0165-4

[44] Sutherland RE, Olsen JS, McKinstry A, Villalta SA, Wolters PJ (2008) Mast cell IL-6 improves survival from Klebsiella pneumonia and sepsis by enhancing neutrophil killing. Journal of Immunology 181: 5598–5605. 10.4049/jimmunol.181.8.5598 PMC2610024 18832718

[45] Piliponsky AM, Chen CC, Rios EJ, Treuting PM, Abrink M, et al (2012) The Chymase Mouse Mast Cell Protease 4 Degrades TNF, Limits Inflammation, and Promotes Survival in a Model of Sepsis. Am J Pathol 181: 875–886. 22901752 10.1016/j.ajpath.2012.05.013 PMC3432424

[46] Berberov EM, Zhou Y, Francis DH, Scott MA, Kachman SD, et al (2004) Relative importance of heat-labile enterotoxin in the causation of severe diarrheal disease in the gnotobiotic piglet model by a strain of enterotoxigenic Escherichia coli that produces multiple enterotoxins. Infect Immun 72: 3914–3924. 15213135 10.1128/IAI.72.7.3914-3924.2004 PMC427467

[47] Argenzio RA, Moon HW, Kemeny LJ, Whipp SC (1984) Colonic compensation in transmissible gastroenteritis of swine. Gastroenterology 86: 1501–1509. 6714576 10.1016/S0016-5085(84)80165-1 PMC7130427

[48] Bailey MT, Coe CL (1999) Maternal separation disrupts the integrity of the intestinal microflora in infant rhesus monkeys. Developmental Psychobiology 35: 146–155. 10461128

